#  Anti-inflammatory action of β-hydroxybutyrate via modulation of PGC-1α and FoxO1, mimicking calorie restriction

**DOI:** 10.18632/aging.101838

**Published:** 2019-02-27

**Authors:** Dae Hyun Kim, Min Hi Park, Sugyeong Ha, Eun Jin Bang, Yujeong Lee, A Kyoung Lee, Jaewon Lee, Byung Pal Yu, Hae Young Chung

**Affiliations:** 1 Department of Pharmacy, College of Pharmacy, Pusan National University, Busan 46241, Korea; 2 Department of Physiology, The University of Texas Health Science Center at San Antonio, TX 78229, USA; 3 Department of Pharmaceutical Sciences, Irma Lerma Rangel College of Pharmacy, Texas A&M Health Science Center, College Station, TX 77843, USA

**Keywords:** β-hydroxybutyrate, aging kidney, calorie restriction, inflammation, FoxO1

## Abstract

β-Hydroxybutyrate (HB) is a ketone body used as an energy source that has shown anti-inflammatory effects similar to calorie restriction (CR); Here, PGC-1α, an abundantly expressed co-factor in the kidney, was reported to interact with both FoxO1 and NF-κB although the definitive interactive mechanism has not yet been reported. In this study, we investigated whether renal aging-related inflammation is modulated by HB. We compared aged rats administered with HB to calorie restricted rats and examined the modulation of FoxO1 and the NF-κB pathway through interactions with PGC-1α. We found that in aged rats treated with HB, pro-inflammatory signaling changes were reversed and showed effects comparable to CR. As FoxO1 and its target genes catalase/MnSOD were upregulated by HB treatment and PGC-1α selectively interacted with FoxO1, not with NF-κB, and ameliorated the renal inflammatory response. These findings were further confirmed using FoxO1 overexpression and siRNA transfection *in vitro*. Our findings suggest that HB suppressed aging-related inflammation as a CR mimetic by enabling the co-activation and selective interaction between FoxO1 and PGC-1α. This study demonstrates the potential therapeutic role of HB as a CR mimetic, which ameliorates inflammation by a novel mechanism where FoxO1 outcompetes NF-κB by interacting with PGC-1α in aging kidneys.

## Introduction

Age-related changes in kidney function and structure, and their relationships have been extensively recognized recently [1]. The effects of aging on the kidneys are more dramatic than any other organ because of diverse factors that can accelerate the changes [1]. Epidemiologic studies have suggested that aging-related renal changes may be associated mainly with systemic hypertension, diabetes, dyslipidemia, and other environmental causes such as smoking [2,3]. With aging, most subjects show a progressive functional decline that includes alterations in inflammatory signaling. A number of interventions for chronic kidney inflammation and fibrosis have been proven to be effective, and it has been reported that caloric restriction (CR) greatly reduces several risk factors related to kidney aging [4]. For example, kidney aging indicators such as glomerular enlargement and mitochondrial abnormality have been delayed or even reversed by CR [4]. Similarly, other researchers have demonstrated that CR attenuates most of the known age-related markers in the kidney, such as glomerular basement membrane thickness, mitochondrial mass in complex proximal tubules, and autophagy markers [5]. However, the molecular mechanism on how CR can delay age-related kidney inflammation needs to be further investigated. Understanding the mechanisms underlying CR is of great importance as this could lead to the identification of new therapeutic targets for age-associated inflammatory diseases.

Ketone bodies include acetoacetate, β-hydroxybutyrate (HB), and acetone, which are three water-soluble molecules that are produced in the mitochondrial matrix of liver from fatty acids and serve as a circulating energy source for tissues during fasting periods [6]. The majority of the ketone bodies are produced in the liver, while smaller amounts are also produced in other tissues [7]. Ketone bodies mediate the neuroprotective effects of CR and also mimic the life-span extension effect of CR in *C. elegans* [8]. Some studies have shown that ketone bodies circulating in low concentration have anti-inflammatory effects. HB has been reported to exert antioxidant effects by upregulating the transcription of antioxidant genes including manganese superoxide dismutase (MnSOD) and forkhead transcription factors 3 (FoxO3) [9]. Although many studies have suggested molecular mechanisms underlying HB and anti-inflammatory effects, their relation to the presumed anti-inflammatory signaling of FoxO remains unknown.

FoxO proteins are well-documented targets and regulators of metabolism, cell cycle, cell death, and oxidative stress response [10]. One member of this family, FoxO1, plays important roles in anti-inflammatory functions [11]. Based on previous reports, it has been demonstrated that one of the key mechanisms by which FoxO is regulated is phosphorylation. In response to insulin or growth factors, for instance, FoxO proteins are phosphorylated by protein kinase B (PKB, also known as Akt), a downstream kinase of phosphatidylinositol 3-kinase (PI3K), which results in the translocation of FoxO from the nucleus to the cytoplasm [12]. More intricate interactions between Akt and FoxO in the cellular regulatory mechanisms have been recently revealed. For instance, in yeast, a mutation in Sch9, which is homologous to Akt, extends lifespan [13], and a mutation of the insulin receptor that decreases activity in the insulin/IGF-1-like pathway increases the longevity of fruit flies [14] and mice [15]. It is interesting to note that these lifespan-extending mutations are associated with increased resistance to oxidative stress, which is partly mediated by the increased expression of antioxidant genes [16]. In addition, other studies reported that the pro-inflammatory nuclear transcription factor (NF-κB) activity is enhanced in the heart, kidney, and brain tissues during loss of tissue homeostasis in the aging process [17].

Several recent studies have investigated effects of aging on the modulation of the redox-sensitive transcription factor NF-κB. The age-related activation of NF-κB has been linked to increased oxidative stress during aging, which has been shown to be effectively suppressed by CR [18]. NF-κB controls the expression of various gene products that affect important cellular processes, such as inflammation, adhesion molecules, cell cycle, angiogenesis, and apoptosis [18]. Transcriptionally active NF-κB is typically a heterodimeric protein complex composed of p50 and RelA/p65. It has been reported that NF-κB and FoxO1 are both involved in the PI3K/Akt age-related inflammatory signaling pathway. It has been proposed that age-related phosphorylation of FoxO1 induces NF-κB activation through the repression of anti-oxidant gene expression. Furthermore, the regulation of age-associated pro-inflammatory genes has been hypothesized to be modulated by the anti-aging action of CR [11].

In the present study, we showed that chronic inflammation in the kidney is a major contributor to age-related changes. The kidney is only second to the heart in terms of mitochondrial abundance [19]. PGC-1α, which is enriched in renal tubules and important for stress resistance in the brain, heart, and other metabolically active organs [20], regulating oxidative metabolism in the renal epithelium to affect overall kidney homeostasis. PGC-1α interacts with FoxO1 and coactivators of FoxO1-dependent genes [21]. Furthermore, PGC-1α counteracts inflammation by reducing the activity of NF-κB [22] as well as leading to a decrease in the phosphorylation of the NF-κB family member p65, thereby reducing its transcriptional activation [23]. However, the molecular interactions among PGC-1α, NF-κB, and FoxO1 in age-related inflammatory responses and anti-inflammatory effects induced by CR have not been reported.

In the present study, we investigated the anti-inflammatory effect of HB, as a mimetic of CR, in aged kidneys and assessed the potential changes in PGC-1α and its competitive interactions with FoxO1 and NF-κB. We demonstrated that HB has a mechanism similar to CR, induced co-activation of FoxO1/PGC-1α through the suppression of the PI3K/Akt pathway, resulting in the inactivation of NF-κB/PGC-1α. This study suggests that HB might be a potential therapeutic candidate for suppressing renal aging-related inflammation.

## RESULTS

### HB ameliorated age-related hyperinsulinemia

**Figure 1:**
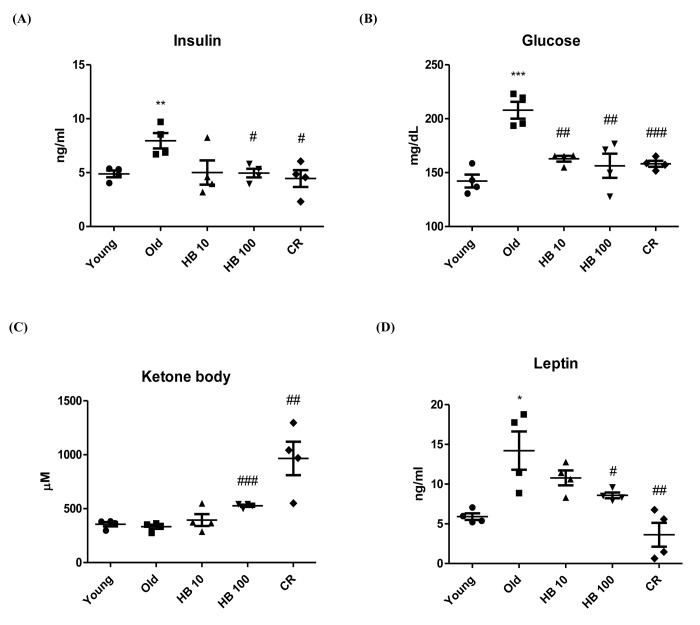
**Ameliorated serum in β-hydroxybutyrate (HB)-treated aged rats.** HB was administered to aged rats (n = 4 each). (**A**) Serum insulin, (**B**) glucose levels, (**C**) Ketone body, and (**D**) Leptin were measured after 30 days of HB treatment. ^##^p < 0.01, ^###^p < 0.001 vs. Young; ^*^p < 0.05, ^**^p < 0.01, ^***^p < 0.001 vs. Old.

To investigate whether HB modulated age-related glucose and insulin changes, we measured serum glucose and insulin levels in relation to aging-related kidney function in aging. The food intake and body weight of rats in young and old groups were similar (Fig. S1). To investigate the effect of HB on aging-related kidney insulin resistance, aged rats were used. Aging resulted in significantly elevated plasma insulin and fasting glucose levels (Fig. 1A, B). The glucose and insulin levels were significantly decreased in the HB-treated and CR group compared with aged rats. However, CR or HB-treated groups (100 mg/kg) had increased ketone body levels in the serum (Fig. 1C). Furthermore, leptin levels decreased in the CR or HB-fed groups compared with the levels in aged rats (Fig. 1D).

### Effects of HB on the expression of FoxO1-dependent target genes MnSOD and catalase

**Figure 2:**
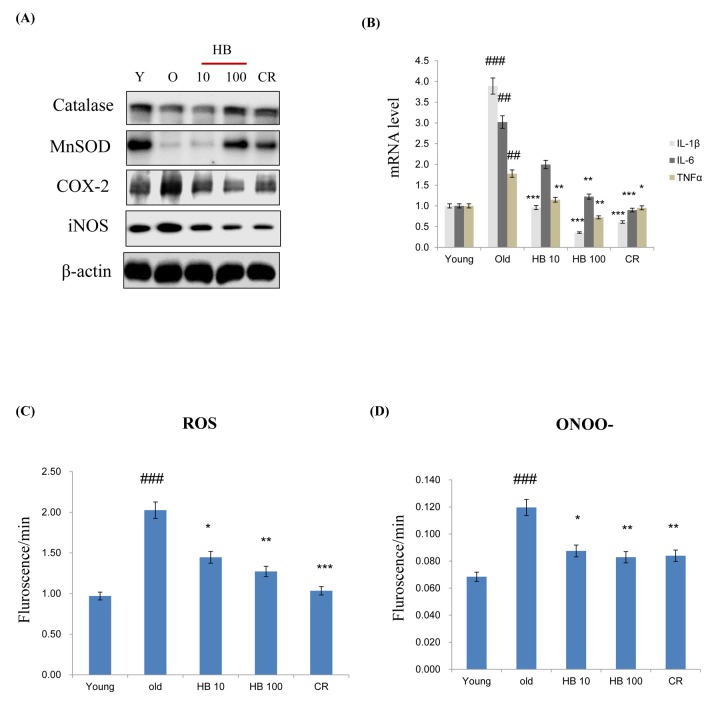
**β-hydroxybutyrate (HB) decreases the expression of inflammatory proteins and activates FoxO1 expression in the kidney of aged rats.** (**A**) Western blotting was performed to examine the protein levels of catalase, MnSOD, COX-2, and iNOS in the kidney of aged rats treated with HB and a caloric restriction (CR). Three independent experiments were performed, and similar results were obtained. (**B**) Expressions of genes encoding cytokines such as IL-1β, IL-6, and TNFα were analyzed using qRT–PCR (n = 4 each). Results were normalized to the GAPDH levels. ^##^p < 0.01, ^###^p < 0.001 vs. Young; ^*^p < 0.05, ^**^p < 0.01, ^***^p < 0.001 vs. Old. (**C**) Levels of reactive oxygen species and (**D**) ONOO^-^ assessed in the kidney of aged rats treated with HB and CR. Three independent experiments were performed, and similar results were obtained. The data are expressed as the mean ± SEM (n = 4). ^###^p < 0.001 vs. Young; ^*^p < 0.05, ^**^p < 0.01, ^***^p < 0.001 vs. Old.

MnSOD and catalase are two major antioxidant enzymes that play a role in the protection against oxidative stress through the metabolism of ROS. In order to examine whether antioxidant protein expression levels were increased with aging and decreased by HB treatment as a CR mimetic in the kidney, western blotting was performed. The expression levels of both MnSOD and catalase were decreased, while it was reversed by HB treatment. Next, to examine age-related changes in target genes of proinflammatory NF-κB, western blot analysis using cytosolic extracts was performed. The results showed that the protein levels of COX-2 and iNOS were increased with aging in control rats, while they remained low and unchanged in the HB-fed and CR groups (Fig. 2A). However, HB treatment and CR were shown to decrease mRNA levels of IL-1β, IL-6, and TNFα compared to the levels in untreated, aged rats (Fig. 2B). In addition, HB treatment was accompanied by a decrease of ROS (Fig. 2C) and ONOO^-^ (Fig. 2D). These data indicated a relationship between FoxO1 phosphorylation and NF-κB activity during the aging process. HB reduced aging-related inflammation via the activation of FoxO1, leading to a reduction of inflammation through the anti-aging effect of HB, which is comparable to the CR effect.

### **Interaction of PGC-1**α **between FoxO1 and NF-κB** **in aging**

**Figure 3:**
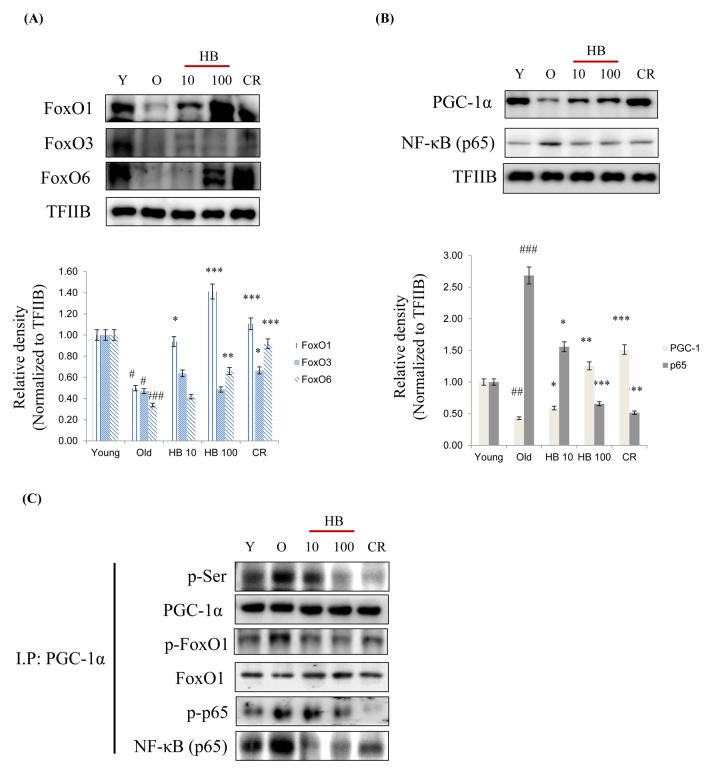
**Caloric restriction (CR) and β-hydroxybutyrate (HB) increases FoxO1 activation during aging.** Western blot analyses for renal nuclear (**A**) FoxO1, FoxO3, and FoxO6 as well as (**B**) PGC-1α and p65 were performed on nuclear proteins from rats treated with Young, Old, HB, and CR. Western blot results from 3 independent experiments were quantified by densitometry. ^#^p < 0.05, ^##^p < 0.01, ^###^p < 0.001 vs. Young; ^*^p < 0.05, ^**^p < 0.01, ^***^p < 0.001 vs. Old. (**C**) Nuclear extracts were prepared from kidneys from young and aged rat. Immunoprecipitation assay showed PGC-1α was physically associated with p-serine, p-FoxO1, FoxO1, p-p65, and p65.

Because FoxO transcription factors play a central role in the regulation of stress response [24], we investigated their modifications during aging. As shown in Fig. 3A, the expressions of three FoxO family members, FoxO1, FoxO3, and FoxO6 decreased during aging, but this reduction was effectively counteracted by CR and HB. Furthermore, NF-κB phosphorylation levels were higher in old rats than in young rats, whereas CR and HB treated old rats showed comparatively lower NF-κB levels (Fig. 3B). PGC-1α levels were also lower in old rats, and CR and HB animals showed higher PGC-1α levels than the same aged animals (Fig. 3B). To identify the mechanism responsible for the inability of FoxO1 to undergo subcellular redistribution, we examined the association between FoxO1 and PGC-1α, a scaffold protein known to bind FoxO and modify its transcriptional activity [25]. We found decreased association between FoxO1 and PGC-1α by increased phosphorylation of serine during aging in the control animals and an increased interaction between FoxO1 and PGC-1α in the CR and HB-treated animals. Conversely, HB and CR were observed to suppress the age-related increase of phosphorylated serine of PGC-1α (Fig. 3C). These data indicate the interaction between FoxO1 and PGC-1α decreases in an aging-dependent manner, and can be reversed by CR and HB.

### Effects of HB on insulin signaling

The binding of insulin and growth factors to specific receptor tyrosine kinases activates PI3K and serine-threonine kinase Akt. Akt promotes cell survival and proliferation in part by directly phosphorylating and inhibiting members of FoxO [12].

**Figure 4:**
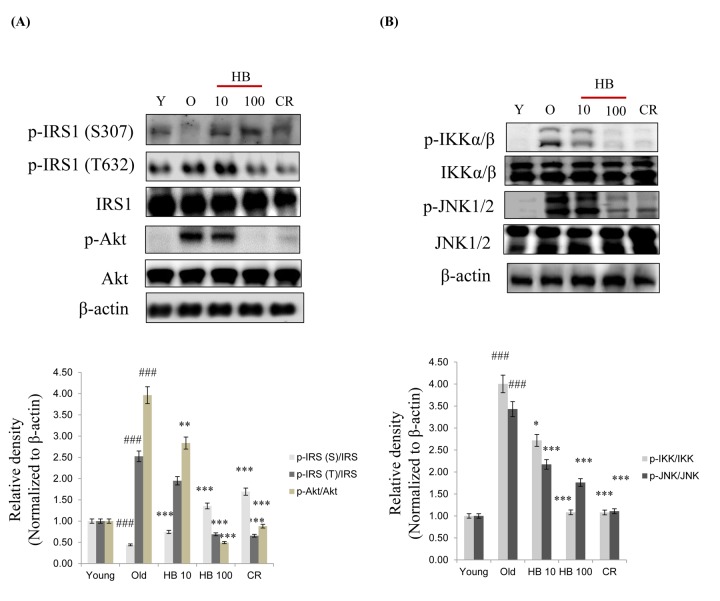
**β-hydroxybutyrate (HB) decreases the expression of insulin signaling in aged rats.** (**A**) Western blotting was performed to examine the protein levels of p-IRS-1 (Ser307), p-IRS-1 (Tyr632), IRS, p-Akt, and Akt as well as (**B**) p-IKK, IKK p-JNK, and JNK in the kidney of aged rats treated with HB. Three independent experiments were performed, and similar results were obtained. Western blot results from 3 independent experiments were quantified by densitometry. ^###^p < 0.001 vs. Young; ^*^p < 0.05, ^**^p < 0.01, ^***^p < 0.001 vs. Old.

We therefore analyzed signaling molecules in the insulin pathway leading to Akt activation and increased insulin levels. Insulin activates PI3K and its downstream target Akt. Therefore, to determine whether a change in FoxO phosphorylation is caused by the activation of the PI3K/Akt pathway, phosphorylated Akt (the active form of Akt) was investigated. Although total Akt amount did not change, aging increased phosphorylation of Akt at Ser473 (Fig. 4). Conversely, HB and CR were observed to suppress the age-related increase of phosphorylated serine-Akt and tyrosine-IRS1 (Fig. 4A). However, Insulin signaling also suppressed the serine phosphorylation of the insulin receptor substrate-1 (IRS-1) and thus increased the tyrosine phosphorylation of IRS-1 as well as the serine phosphorylation of Akt in aging. On the other hand, HB and CR attenuated tyrosine phosphorylation of IRS-1 and serine phosphorylation of Akt (Fig. 4A). Next, we measured the activation of NF-κB signaling during aging. However, to determine whether a change in NF-κB phosphorylation is caused by activation of the IKK and JNK pathways, the phosphorylation of IKK and JNK was investigated. HB and CR were observed to suppress the age-related increase of phosphorylated IKK and JNK (Fig. 4B). These data suggest that PI3K/Akt signaling, upregulated by increased insulin levels might be associated with FoxO phosphorylation during the aging process, while CR or HB can reverse these phenomena.

### Verification of enhanced FoxO1 inactivation in insulin-treated cultured cells

**Figure 5:**
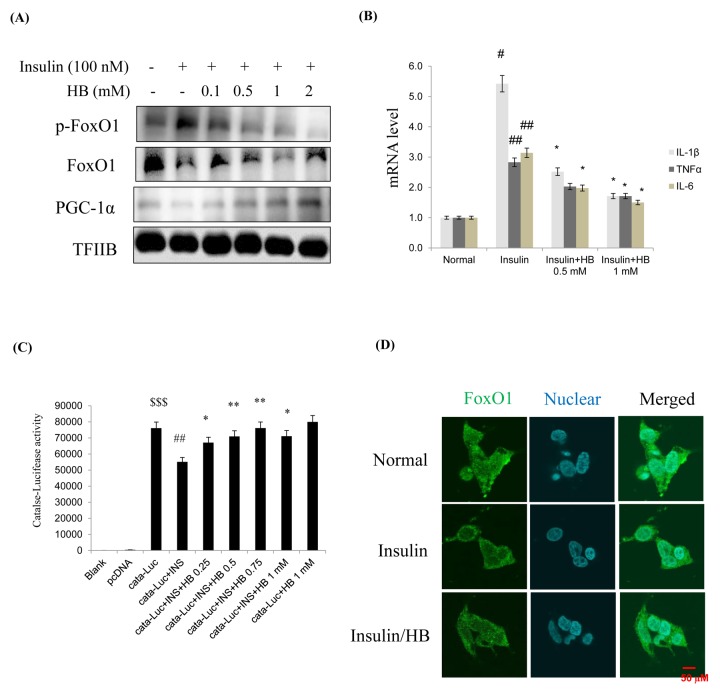
**β-hydroxybutyrate (HB) regulates insulin-induced expression of FoxO1.** (**A**) Levels of p-FoxO1, total FoxO1, and PGC-1α were noticeably diminished after treatment with 0.1–2 mM HB for 3 h, followed by incubation with or without 100 nM of insulin for 2 h. (**B**) Expressions of genes encoding cytokines such as IL-1β, TNFα, and IL-6 were analyzed using qRT–PCR (n = 3 each). Results were normalized to GAPDH mRNA levels. ^#^p < 0.05, ^##^p < 0.01 vs. Normal; ^*^p < 0.05, vs. insulin treated group. (**C**) HEK293T cells were transiently transfected with a catalase and catalase-containing plasmid linked to the luciferase gene, pre-incubated with HB (0.25–1 mM) for 4 h, and then treated with insulin for 24 h. Results are presented in relative luminescence units (RLU). Results were obtained using one-factor ANOVA: ^$$$^p<0.001 vs. pcDNA transduced cells; ^##^p<0.01 vs. catalase-luciferase transduced cells; ^*^p<0.05, ^**^p<0.01 vs. insulin with catalase-luciferase transduced cells. (**D**) HEK293T cells were pretreated with or without 0.5 mM of HB for 3 h and then treated with insulin (100 nM) for 10 min. Cells were immunostained using rabbit anti-FoxO1 antibody followed by IgG conjugated with fluorescein isothiocyanate (green). Bar = 50 µm.

When HEK293T cells were treated with insulin (100 nM for 15–120 min), anti-inflammatory genes showed a remarkable decrease due to activation of Akt (Fig. S2). Cells were pretreated with HB and then treated with or without 100 nM insulin in media from 24 h. FoxO1 levels and target genes were analyzed by western blotting. As shown in Fig. 5A, treatment with 100 nM insulin suppressed FoxO1 levels in nucleus, while nuclear FoxO1 was markedly increased by HB treatment. This indicated that insulin enhanced the phosphorylation of FoxO1 on Ser256, which could be reversed by HB. Recent evidence indicates that mammalian FoxO upregulates the transcription of free radical scavengers MnSOD and catalase, which have a protective effect against oxidative damage in human cells [26]. The levels of catalase and MnSOD were reduced in HEK293T cells by insulin treatment. These results strongly suggest that constitutive activation of Akt increases the phosphorylation of FoxO1, thereby downregulating catalase and MnSOD. Furthermore, HB reduced catalase, but not MnSOD levels (Fig. S3). In addition, HB increased cell viability and suppressed insulin, suggesting that HB regulates toxicity under insulin conditions (Fig. S4). In addition, HB treatment resulted in decreased mRNA levels of IL-1β, TNFα, and IL-6 compared to the insulin-treated group (Fig. 5B).

The transcriptional activities of FoxO family proteins have been reported to be increased when insulin levels were reduced [27]. We hypothesized that FoxO1 targets the catalase gene for transactivation; therefore, we examined the ability of FoxO1 to stimulate catalase expression in HEK293T cells. To examine whether FoxO1 directly regulates catalase transcriptional activity and whether HB activates this process, we performed a catalase luciferase assay after insulin treatment. Insulin significantly decreased catalase activity, and HB treatment increased catalase activity in HEK293T cells. (Fig. 5C). These observations support the idea that FoxO1 targets the catalase gene for transactivation, and thus contributes to the regulation of insulin. As shown in Fig. 5D, treatment with 100 nM insulin induced a remarkable shift of FoxO1 from the nucleus to the cytoplasm as determined by immunostaining, which was inhibited by HB. These data suggest that insulin might be associated with FoxO1 phosphorylation, leading to cytokine expression, while HB reversed these phenomena.

### Verification of enhanced NF-κB activation in insulin-treated cultured cells

**Figure 6:**
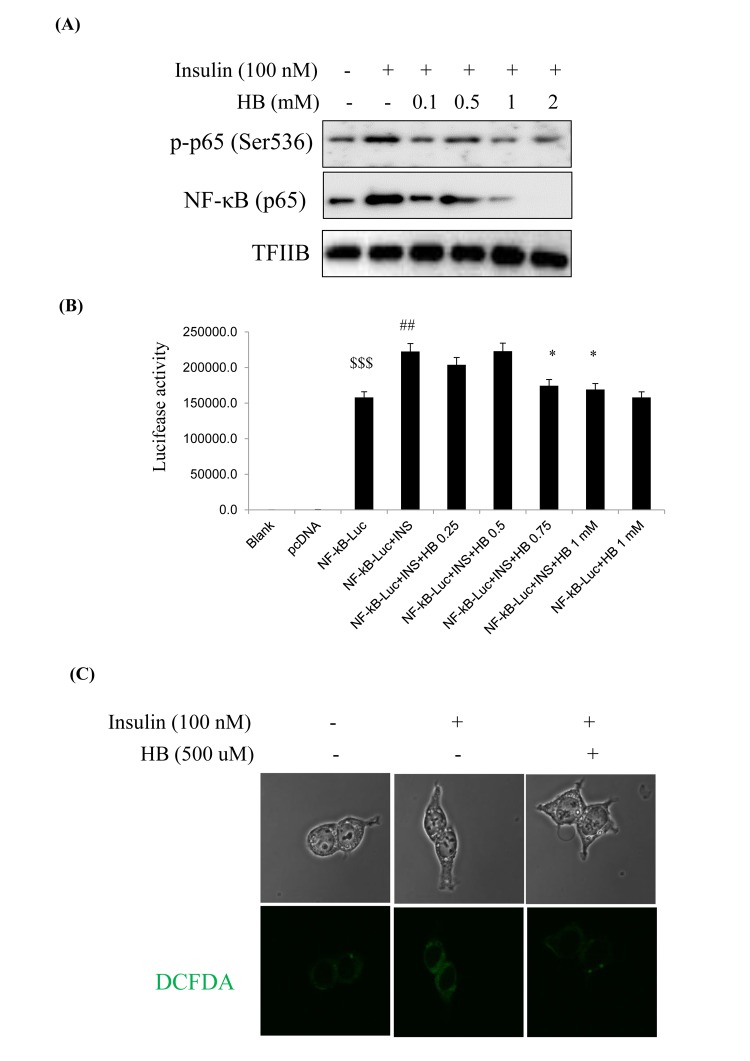
**β-hydroxybutyrate (HB) regulates insulin-induced inflammation.** (**A**) Levels of p-p65 and total p65 noticeably diminished after treatment with 0.1–2 mM HB for 3 h, followed by incubation with or without 100 nM insulin for 2 h. (**B**) HEK293T cells were transiently transfected with a NF-κΒ and NF-κΒ-containing plasmid linked to the luciferase gene, pre-incubated with HB (0.25–1 mM) for 4 h, and then treated with insulin for 24 h. Results are presented in relative luminescence units (RLU). Results were obtained using one-factor ANOVA: ^$$$^p<0.001 vs. pcDNA transduced cells; ^##^p<0.01 vs. NF-κB-luciferase transduced cells; ^*^p<0.05 vs. insulin with NF-κB-luciferase transduced cells. (**C**) Confocal laser microscopy analysis of intracellular ROS levels using 2’,7’-dichlorodihydrofluorescein diacetate. Cells were incubated with 100 nM insulin for 2 h after pretreatment with HB for 3 h. Panel 1, normal; panel 2, 100 nM insulin; panel 3, 100 nM insulin + 0.5 mM HB.

We examined the relationship of NF-κB and the insulin signaling pathway in HEK293T cells. Cells were pretreated with HB and then treated with or without 100 nM insulin in the media for 24 h. NF-κB levels and target genes were analyzed by western blotting. As shown in Fig. 6, phosphorylated p65 and NF-κB (p65) levels were increased in the nucleus in response to the insulin challenge, which were reduced by HB (Fig. 6A).

To investigate the role of NF-κB in insulin-induced oxidative stress, we examined the expressions of proinflammatory genes such as COX-2 and iNOS. COX-2 and iNOS levels were shown to increase with insulin, while HB treatment markedly reduced COX-2 and iNOS levels (Fig. S5). These results strongly suggest that constitutive activation of Akt increased the phosphorylation of NF-κB, leading to increased ROS that promoted oxidative stress-induced inflammation via NF-κB pathway. We examined the activation of NF-κB in HEK293T cells. FoxO1 was shown to be associated with the activity of insulin at the NF-κB promoter, as determined by luciferase activity in HEK293T cells. Insulin associated with the NF-κB promoter DNA in HEK293T cells, thereby enhancing the effect of NF-κB activity. On the other hand, NF-κB promoter activity was reduced in HB treated cells (Fig. 6B). As shown in Fig. 6C, ROS levels were increased in insulin-treated cells as compared to untreated control, which was reversed by treatment with HB.

### Effect of HB on FoxO1 phosphorylation through the PI3K/Akt pathway

When insulin and growth factors bind to specific receptors, tyrosine kinases activate PI3K and the serine-threonine kinase Akt. Akt promotes cell survival and proliferation in part by directly phosphorylation and inhibiting members of the FoxO subfamily of forkhead transcription factors. We examined the relationship of FoxO1 via the insulin signaling pathway in HEK293T cells.

Insulin exerts its action activating PI3K and its downstream target Akt. Therefore, to determine whether the change in FoxO phosphorylation was caused by the activation of the PI3K/Akt pathway, phosphorylated Akt was investigated. Although total Akt amounts were not changed, phosphorylated Akt in Ser473 was shown to increase with insulin (Fig. S6A). However, insulin treatment also decreased serine phosphorylation of insulin resistance substrate-1 (IRS-1) and thus increased tyrosine phosphorylation of IRS-1. Conversely, HB treatment was observed to suppress the increase of phosphorylated IRS/Akt signaling (Fig. S6A). To further examine the effect of HB on hyperinsulinemia, we studied the interaction of FoxO1, PGC-1α, and NF-κB in HEK293T cells using insulin-treated cells. The IP results showed that the interaction of FoxO1, PGC-1α, and NF-κB was reversed by HB (Fig. S6B). These data suggest that upregulated PI3K/Akt signaling by increased insulin levels might be associated with FoxO1 phosphorylation and that HB treatment reversed these phenomena.

**Figure 7:**
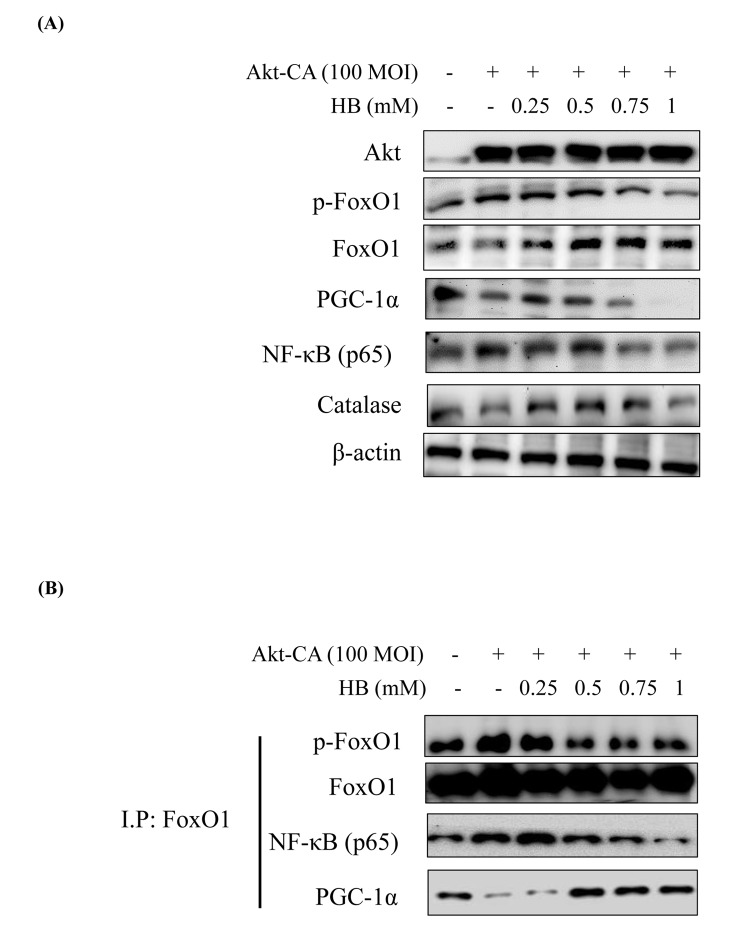
**β-hydroxybutyrate (HB) regulates the activation of FoxO1 phosphorylation through the PI3K/Akt pathway.** HEK293T cells were grown to 80% confluency in 100 mm dishes in DMEM medium, pre-treated (1 day) with or without CA-Akt (100 MOI), and then treated with 0.25–1 mM HB. (**A**) Cells were pre-transduced in the absence or presence of CA-Akt vector (100 MOI), and analyzed by western blotting. (**B**) Immunoprecipitation showed that FoxO1 was physically associated with phosphorylated FoxO1, FoxO1, p65, and PGC-1α after stimulation with HB (0.25–1 mM) in the absence or presence of CA-Akt (100 MOI).

Several authors have suggested that members of the FoxO family are regulated by the PI3K/Akt pathway. More specifically, Akt, a key downstream effector of PI3K, is believed to phosphorylate FoxO proteins directly or to promote their phosphorylation by other kinases [12]. Consequently, Akt suppress PGC-1α activity by Ser570 phosphorylation of PGC-1α [28]. We examined the PI3K/Akt pathway and its effect on FoxO1 phosphorylation utilizing constitutively active Akt (CA-Akt) and measured FoxO1 phosphorylation by Akt. We found reduction in FoxO1 activity when the concentration of Akt was increased. However, PGC-1α expression was suppressed at various concentrations of the Akt vector (Fig. S7). Accordingly, we treated HEK293T cells with CA-Akt (100 MOI) for 1 day, the cells were then incubated with HB for 4 h, and then FoxO1, PGC-1α, NF-κB, and Akt levels were assessed. As shown in Fig. 7A, in HEK293T cells, FoxO1 and PGC-1α were suppressed in CA-Akt. Otherwise, FoxO1 and PGC-1α were increased, and these decreased NF-κB by HB treatment. These results indicate that the phosphorylation of FoxO1 in kidney cells was associated with the Akt activation, and HB inhibited Akt activation.

To further examine the effect of HB on Akt activation, we studied the interaction of FoxO1, PGC-1α, and NF-κB in HEK293T cells using insulin-treated cells. The IP results showed that the interaction of FoxO1, PGC-1α, and NF-κB was reversed by HB (Fig. 7B). These data indicate that FoxO1 and NF-κB interact with PGC-1α in an insulin-dependent manner and that HB interferes with the interaction between FoxO1 and PGC-1α.

### Effects of HB on FoxO1-knockdown kidney cells

**Figure 8:**
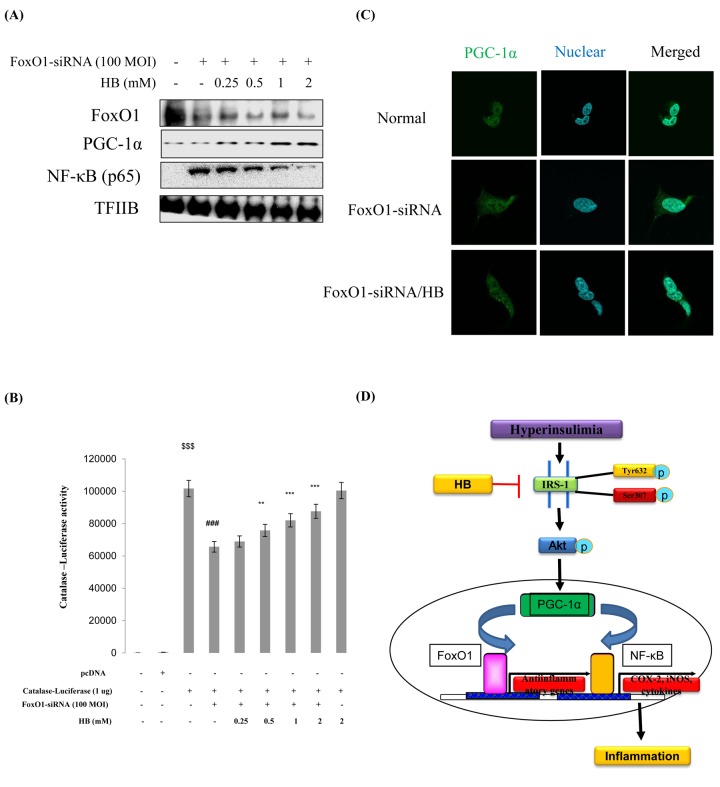
**Effect of β-hydroxybutyrate (HB) on FoxO1-dependent gene expression after FoxO1 knockdown.** Western blot analysis was used to assess protein levels in FoxO1 siRNA-treated HEK293T cells. (**A**) FoxO1, PGC-1α, and NF-κB protein levels in cells pretreated for 3 h with HB in the absence or presence of FoxO1 siRNA-transfected cells (200 MOI) for 1 day. (**B**) HEK293T cells were transiently transfected with a catalase-containing plasmid linked to the luciferase gene, pre-incubated with FoxO1-siRNA (100 MOI) for 24 h, and then treated with HB for 4 h. Results are presented in relative luminescence units (RLU). Results were obtained using one-factor ANOVA: ^$$$^p<0.001 vs. pcDNA transduced cells; ^###^p<0.001 vs. catalase-luciferase transduced cells; ^**^p<0.01, ^***^p<0.001 vs. FoxO1-siRNA with catalase-luciferase transduced cells. (**C**) HEK293T cells were pretreated with or without 0.5 mM HB for 3 h and then treated with FoxO1-siRNA (200 MOI) for 24 h. Cells were immunostained using rabbit anti-PGC-1α antibody followed by IgG conjugated with fluorescein isothiocyanate (green). Bar = 50 µm. (**D**) A possible mechanism underlying the effect of HB regulate NF-κB through interaction of FoxO1 and PGC-1α in aging.

We examined PGC-1α and NF-κB genes expression in FoxO1 virus-transduced HEK293T cells. Cells were treated with or without different concentrations (100 and 200 MOI) of FoxO1-CA virus. As shown in Fig. S8, treatment with FoxO1 induced PGC-1α levels. Otherwise, NF-κB was reduced by FoxO1 activation. These data collectively indicate that proinflammatory NF-κB suppresses FoxO1 activation. To investigate the effect of HB and the relation of FoxO1 and inflammation under FoxO1 knockdown conditions, we examined the expression of inflammatory markers COX-2 and iNOS. The results showed that the HB-treated groups had higher levels of FoxO1 and PGC-1α than FoxO1 siRNA-knockdown cells (Fig. 8A). In addition, to determine the effect of HB on inflammation, we measured COX-2 and iNOS by western blotting. The results showed that HB decreased the expression of the inflammatory proteins compared with FoxO1 siRNA-knockdown cells (Fig. S9). Next, we examined the ability of HB to stimulate catalase and MnSOD expression in HEK293T cells. FoxO1 was shown to be associated with activity at the catalase promoter, as determined by a luciferase assay in FoxO1-siRNA knockdown HEK293T cells (Fig. 8B). As shown in Fig. 8C, treatment with FoxO1-siRNA induced a remarkable shift of PGC-1α from the cytoplasm to the nucleus as determined by immunostaining, while HB inhibited the translocation of PGC-1α from the nucleus to the cytoplasm.

## DISCUSSION

Based on the current studies in the literature, the application of exogenous ketone bodies, including HB, appears to be a viable strategy for increasing tolerance to the ketogenic diet, although its underlying mechanism is not well known. In the present study, we reported the critical role of HB, which plays an anti-inflammatory role similar to CR in the renal aging process via the co-activation of FoxO1/PGC-1α and inactivation of NF-κB in the PI3K/Akt pathway. We first confirmed that serum insulin levels increased with aging, while HB treatment reversed this age-related effect (Fig. 1). While investigating the mechanism of the hyperinsulimia axis leading to the proinflammatory response in kidney, we found a unique function of PGC-1α, which selectively interacted with either FoxO1 or NF-κB to determine differential responses that resulted in anti-inflammatory or pro-inflammatory responses, respectively. In addition, we have shown that PI3K/Akt positively regulates NF-κB in HEK293T cells and during aging via regulating the interaction of FoxO1 and PGC-1α, and that this association might be mediated at least in part by FoxO1, which regulates cellular ROS levels by regulating catalase (Fig. 2 and 3). Our study provides significant findings demonstrating that HB could be used as a potential CR mimetic, which has beneficial effects of anti-inflammation in the kidney as demonstrated *in* *vivo* and *in vitro*.

The positive effects of CR in the aging process were well reviewed, and potential therapeutic application of CR has been also reported in aging-related disorders such as obesity, insulin resistance, type 2 diabetes, atherosclerosis, and cancer [29,30]. Ketone bodies, well-known CR mimetic, are metabolic products that are released into the blood circulation produced mostly from the liver and utilized in extrahepatic tissues as an energy source in fasting state. HB exerts pleiotropic health benefits mainly via ketogenesis, a process of ketone body production. Ketogenesis begins within 24 h of fasting through gluconeogenesis [31]. Recent study showed that CR increased the HB concentration when compared to feeding *ad libitum* [9]. In addition, others have shown that a ketogenic diet has therapeutic effects on diseases related to insulin resistance as well as diseases resulting from free radical damage, and hypoxia [32]]. In addition, HB contributed to the anti-inflammatory effect in rapid weight loss [33]]. These observations provide evidence to HB-mediated beneficial health effects as CR mimetics, which include the suppressive action of age-related inflammation.

We have shown that expression of antioxidant genes MnSOD and catalase were upregulated upon HB treatment in age-related kidney (Fig. 2). This upregulation was derived from increased expression of key anti-oxidant transcription factors FoxO1 and PGC-1α (Fig. 3). Based on previous reports, PGC-1α protects against oxidative stress by increasing the expression of various antioxidant defense enzymes including catalase, copper/zinc superoxide dismutase, manganese superoxide dismutase, and glutathione peroxidase [34]. PGC-1α interacts with FoxO1 and coactivates FoxO1-dependent gene expression [35]. FoxO are well-known transcription factors for longevity, which can regulate directly or indirectly the inhibition of proinflammatory factor NF-κB [36], and it enhances cellular defenses against oxidative stress during aging [37].

Type 2 diabetes induced by insulin resistance has been suggested as a cause of accelerated aging [38]. In an insulin resistant state, hyperinsulinemia-induced receptor tyrosine kinase activation commonly leads to activation of PI3K/Akt signaling, and thus activates proinflammatory NF-κB in kidney [39,40]. Based on our results, insulin activates the PI3K/Akt pathway, resulting in the phosphorylation and inactivation of FoxO1 and PGC-1α, which prevents these factors from translocating into the nucleus and thereby suppressing the transcription of proinflammatory cytokines. Furthermore, we have shown that this signaling induction was reversed upon HB treatment. In HB treated conditions, FoxO1 was dephosphorylated and transported into the nucleus to associate with PGC-1α, which is also in a dephosphorylated active form (Fig. 4 and 5). Initial evidence showing that PI3K controls FoxO activity came from genetic studies performed with the nematode, *C. elegans*, where PI3K suppressed the function of DAF-16 or FoxO [41]. This PI3K-FoxO signaling was found to be critical for metabolic control and cell survival [42]. FoxO proteins bind to insulin responsive element **(**IRE) in the proximal promoter and activate target genes involved in cell survival, cell cycle, DNA repair, and insulin sensitivity [42]. In relation to immunoregulatory function, Lin et al. [43], reported that activated FoxO downregulates insulin-induced ROS production and increased the NF-κB transcription factor in the proinflammatory signaling pathway, which was also confirmed in our study (Fig. 6). NF-κB, a ubiquitous proinflammatory transcription factor known to be activated by a wide variety of stimuli, including ROS and oxidative stress [44], was identified as a functional target of Akt [45]. As NF-κB is activated in response to oxidative damage through the generation of ROS or perturbations in the cellular redox state, FoxO can either activate or inactivate the NF-κB pathway by modulating ROS production [46].

However, this study has limitations as we primarily explored the acute impact of HB. We administered HB only for 30 days, which is considered a rather short induction period, and the treatment period could have been prolonged further to define the molecular mechanisms representative of the anti-inflammatory effect *in vivo*. In this work, we explored the beneficial effects of HB on kidney inflammation, suggesting that HB supplementation may lead to a restoration of kidney function and alleviate oxidative stress injury in the aging process. The effects of HB appear to be mediated via the activation of the FoxO1/PGC-1α-antioxidant pathway rather than via the proinflammatory NF-κB pathway (Fig. 8D).

In summary, HB reduced oxidative stress and age-related inflammation by leading to an increased interaction between FoxO1 and PGC-1α, resulting in a reduction in inflammation, which is comparable to the effects of CR. We conclude that HB suppresses aging-related inflammation as a CR mimetic via inducing co-activation and selective interaction between FoxO1 and PGC-1α in aged kidneys. This study successfully demonstrated the anti-inflammatory role of HB as a CR mimetic in age-related kidney, which could be potentially useful for developing therapeutic anti-inflammatory strategies.

## MATERIALS AND METHODS

### Materials

All chemical reagents were obtained from Sigma (St. Louis, MO, USA), except where noted. The compound 2’,7’-dichlorodihydrofluorescein (DCFDA) was obtained from Molecular Probes, Inc. (Eugene, OR, USA). Western blotting detection reagents were obtained from Amersham (Bucks, UK). RNAzol^TM^ B was obtained from TEL-TEST Inc. (Friendwood, TX, USA). Antibodies against β-actin, TFIIB, P-Akt, total-Akt, nuclear factor kappa B (NF-κB), p-FoxO1 (S256), FoxO1, PGC-1α, and p-Akt (S473) were obtained from Santa Cruz Biotechnology (Santa Cruz, CA, USA). Horseradish peroxidase-conjugated anti-rabbit IgG, and horseradish peroxidase-conjugated anti mouse IgG antibodies were obtained from Amersham (Bucks, UK). Horseradish peroxidase-conjugated anti-sheep/goat IgG from donkey was purchased from Serotec (Oxford, UK). Polyvinylidene difluoride (PVDF) membranes were obtained from Millipore Corporation (Bedford, MA, USA). (±)-Sodium 3-hydroxybutyrate were purchased from Sigma-Aldrich (ST. Louis, MO, USA), except where noted.

### Animals

Pathogen-free male Sprague Dawley rats were obtained from Samtako (Osan, Korea) and were fed a standard laboratory diet (Superfeed Co., Wonju, Kangwon, Korea) *ad libitum*. Animals at 6 and 24 months of age were used as young and old rats, respectively. There were 4 rats in each experimental group. To estimate the effects of HB on inflammation, HB was injected to old rats by oral gavage (10 and 100 mg/kg/day) for 30 days. This concentration was selected based on a previous study on the regulation of sympathetic nervous system activity by ketone bodies [47]. All animal studies were designed by the Aging Tissue Bank and approved by the Institutional Animal Care Committee of Pusan National University. We followed the guidelines for animal experiments issued by Pusan National University (Approval Number PNU-2012-0088).

### Cell culture system

HEK293T cells were obtained from ATCC (American Type Culture Collection, Rockville, MD, USA). HEK293T cells were cultured in Dulbecco’s modified Eagle medium (DMEM) (Nissui Co., Tokyo, Japan) supplemented with 10% heat-inactivated (56°C for 30 min) fetal bovine serum (Gibco, Grand Island, NY), 233.6 mg/mL glutamine, 72 μg/mL penicillin streptomycin, and 0.25 μg/mL amphotericin B, and adjusted to pH 7.4–7.6 with NaHCO_3_ in an atmosphere of 5% CO_2_. Cells were maintained at 37°C in a humidified atmosphere containing 5% CO_2_.

### Biochemical analysis

Blood samples were collected after the animals in each group had been sacrificed. β-hydroxybutyrate concentrations were determined using a HB detection kit (Stanbio Laboratory, USA). The insulin level was determined using the rat insulin ELISA kit (Shibayagi Co, Japan), glucose, and leptin (Shinyang, South Korea).

### Tissue preparations

Nuclear protein extraction was performed using the method reported by Komatsu (2007). Briefly, renal tissues were homogenized in ice-cold lysis buffer containing 5 mM Tris-HCl (pH 7.5), 2 mM MgCl_2_, 15 mM CaCl_2_, and 1.5 M sucrose, followed by the addition of a 0.1 M dithiothreitol (DTT) and protease inhibitor mixture. After centrifugation (10,500 × g for 20 min at 4°C), the pellet was suspended in an extraction buffer containing 20 mM 2-[4-(2-hydroxyethyl)-1-piperazyl] ethanesulfonic acid (pH 7.9), 1.5 mM MgCl_2_, 0.42 M NaCl, 0.2 mM EDTA, and 25% (v/v) glycerol, followed by the addition of a 0.1 M DTT and protease inhibitor (1 μM pepstatin, 80 mg/L trypsin inhibitor) mixture. The mixture was placed on ice for 30 min. The nuclear fraction was prepared by centrifugation at 20,500 × g for 5 min at 4°C. The post-nuclear fraction was extracted from the kidneys of each rat. Briefly, the renal tissues were homogenized with ice-cold lysis buffer (pH 7.4) containing 137 mM NaCl, 20 mM Tris–HCl, 1% Tween 20, 10% glycerol, 1 mM PMSF, and protease inhibitor mixture. The homogenate was then centrifuged at 2,000 × g for 10 min at 4°C. The protein concentration in each fraction was determined using a Bio-Rad protein kit (Bio-Rad Laboratories, Hercules, CA, USA).

### Cell lysis

Cells were washed by PBS, and then 1 ml of ice-cold PBS was added. Pellets were harvested at 3,000 rpm at 4°C for 5 min. The pellets were suspended in 10 mM Tris (pH 8.0), with 1.5 mM MgCl_2_, 1 mM DTT, 0.1% Nonidet-40 (NP-40), and protease inhibitors (1 μM pepstatin, 80 mg/L trypsin inhibitor), incubated on ice for 15 min. Nuclei were separated from cytosol by centrifugation at 12,000 rpm at 4°C for 15 min. The supernatants were used as cytosolic fraction and the pellets were resuspended in 10 mM Tris (pH 8.0), with 50 mM KCl, 100 mM NaCl, and protease inhibitors, incubated on ice for 30 min, then were centrifuged at 12,000 rpm at 4°C for 30 min. The resultant supernatants were used as the nuclear fraction.

### Western blot analysis

Homogenized kidney tissues and lysed cell samples (10 μg of protein from each nuclear fraction and 20 μg of protein from each cytosolic fraction) were boiled for 5 min with a gel-loading buffer (pH 6.8, 125 mM Tris-HCl, 4% sodium dodecyl sulfate [SDS], 10% 2-mercaptoethanol, and 0.2% bromophenol blue) in a ratio of 1:1. Equal amounts of protein were separated by SDS-polyacrylamide gel electrophoresis (SDS-PAGE) using 6–17% gels. The gels were subsequently transferred onto an Immobilon-P transfer membrane (Millipore Corp, Bedford, MA, USA). The membrane was immediately placed in a blocking solution (5% non-fat dry milk in TBS-Tween [TBS-T] buffer containing 10 mM Tris, 100 mM NaCl, and 0.1% Tween 20, pH 7.5) at room temperature for 1 h. The membrane was washed in TBS-T buffer for 30 min and incubated with the primary antibody at room temperature for 2 h. After a 30-min wash in TBS-T buffer, followed with either anti-rabbit or anti-mouse IgG HRP-conjugated secondary antibody at 25 ºC for 1 h. After a 40-min wash in TBS-T buffer. Each antigen-antibody complex was visualized using ECL Western Blotting Detection Reagents and detected by chemiluminescence with Sensi-Q 2000 (Lugen Sci., Gyeonggido, Korea). Band densities were determined using ATTO Densitograph Software (ATTO Corporation, Tokyo, Japan) and quantified as a ratio of the TFIIB or β-actin level.

### Luciferase reporter assay

The activity of NF-κB was examined using the luciferase plasmid DNA pTAL-NFκB, which contains a specific binding sequence (5′-ATATACA-3′) linked to a basic promoter element (BD Biosciences Clontech, Mountain View, CA, USA) for FoxO. Catalase activities were estimated using a catalase-Luc vector (a gift from Dr. Dong, University of Pittsburgh, PA, USA), which contained a specific binding sequence for FoxO. Transfection was carried out using FuGENE 6 Reagent (Roche, Indianapolis, IN). Briefly, 1.5 × 10^4^ cells per well were seeded onto 48-well plates. When cultured cells reached about 40% confluency, cells were treated with 0.1 μg DNA/0.5 μL FuGENE 6 complexes in 500 μL normal media (10% serum contained) for 42 h. Subsequently, 0.2 μM of insulin was treated after replacing the media with serum-free media. After an additional incubation for 6 h, the cells were washed with PBS and subjected to Steady-Glo Luciferase Assay System (Promega, Medison, WI, USA). Luciferase activity was measured by a luminometer (GENious, TECAN, Salzburg, Austria).

### Immunoprecipitation (IP) assay

Nuclear extracts were subjected to IP in a buffer containing 40 mM Tris-HCl (pH 7.6), 120 mM NaCl, 20 mM β-glycerophosphate, 20 mM NaF, 2 mM sodium orthovanadate, 5 mM EDTA, 1 mM PMSF, 0.1% NP40 with leupeptin (2 μg/mL), aprotinin (1 μg/mL), and pepstatin A (1 μg/mL). One milligram of nuclear extracts was incubated with 50% slurry of protein A agarose for 30 min at 4°C for preclearing. After incubation, nuclear extracts were centrifuged at 12,000 ×g at 4°C for 5 min. The nuclear extracts were then incubated overnight with the respective antibody at 4°C followed by incubation overnight at 4°C with 50% slurry of protein A agarose. After washing of the immunoprecipitates three times with IP buffer, the immunoprecipitated proteins were analyzed by SDS-PAGE followed by western blotting as described previously.

### Reactive oxygen species (ROS) and ONOO^-^ activity in tissue

ROS generation was measured as previously described (11) in tissue. One hundred and twenty-five μM of 2’,7’-dichlorodihydrofluorescein diacetate (DCFDA) was added to the homogenate with buffer to a final volume of 250 μl. The fluorescence intensity of DCF was measured every 5 min for 1 h using the microplate fluorescence reader TECAN (Salzburg, Austria) with excitation and emission wavelengths of 485 and 535 nm, respectively.

ONOO^-^ activity was measured by monitoring the oxidation of fluorescent dye DHR 123 by a modified method. Briefly, a working solution of 5 μM DHR 123 was loaded onto a 96-well-plate containing 10 μl sample in 50 mM sodium phosphate buffer (pH 7.4) containing 90 mM NaCl and 5 mM KCl. Just before use, 100 μM diethylenetriaminepenta acetic acid (DTPA) was added to the reaction mixture. After 5 min incubation at room temperature, the oxidation status of DHR 123 by ONOO^-^ was measured using a microplate fluorescence spectrophotometer FL 500 (Bio-Tek Instruments, Winooski, VT, USA) with excitation and emission wavelengths of 485 and 530 nm, respectively.

### Analysis of the intracellular ROS levels by confocal laser microscopy

The amount of ROS generated was estimated by assaying the formation of ﬂuorescent DCFDA from the oxidation. Brieﬂy, cells grown to subconﬂuency on glass cover slips were incubated with various agents for 2 h prior to treatment with insulin. Following stimulation with insulin for 3 h, cells were washed with NaCl/Pi, incubated with 10 µM DCFDA for an additional 30 min, and mounted on the microscope stage. Fluorescence images were recorded using a Zeiss LSM 510 laser scanning confocal microscope with excitation at 488 nm and long pass detection at 530 nm.

### Immunostaining

HEK293T cells were seeded at 1 × 10^4^ cells per well in a 12-well plate, incubated for 24 h, fixed in 4% paraformaldehyde solution (15 min at room temperature), washed with PBS buffer, blocked with 3% normal goat serum (Gibco, Grand Island, USA), and immunostained using rabbit anti-FoxO1 or anti-PGC-1α antibody (1:1000 dilution, Santa Cruz, CA) at 4°C overnight. Cells were then washed with TBS and incubated for 3 h in the presence of anti-rabbit IgG labeled with Alexa Fluor 488 (1:200; Invitrogen, CA, USA). Cell nuclei were visualized by immunostaining with Hoechst 33342 (1:1000; Invitrogen), and FoxO1 or PGC-1α was determined by confocal laser scanning microscopy (TCS SP2, Leica, Wetzler, Germany).

### Isolation and quantification of RNA

Rat tissue RNA was homogenized in the presence of RiboEx (GeneAll, Seoul, Korea) using a bead homogenizer (TissueLyser, Qiagen, Hilden, German) according to the manufacturer’s instructions. After being kept at 25°C for 5 min, the sample was centrifuged at 12,000 rpm for 15 min. The RNA pellet was precipitated with isopropanol and centrifuged at 12,000 rpm for 15 min. After the supernatant was removed, the pellet was washed with 75% ethanol. To remove the ethanol supernatant, the pellet was centrifuged at 12,000 rpm for 15 min and dissolved in diethyl pyrocarbonate-treated water. The RNA (2.0 μg) treated with RNase-free water was reverse-transcribed using a cDNA synthesis master kit (GenDEPOT, Barker, Texas, USA). Quantitative polymerase chain reaction (qPCR) was performed using SYBR green real-time master mix (GeneAll, Seoul, Korea) and a CFX Connect System (Bio-Rad Laboratories, Inc., Hercules, CA, USA).

### Cell viability

Cytotoxicity was determined using the MTT assay from Aldrich Chemical Co. (Madison, WI, USA). Cells were seeded onto 96-well plates and incubated overnight to adhere. The cells were then treated with insulin (100 nM), with or without HB, for 3 h. At the end of the treatment period, the MTT reagent dissolved in PBS was added to the medium (final concentration 0.5 mg/mL), and the plates were incubated in the dark for 1 h. After the incubation, the supernatant was removed, and the formazan crystals formed were dissolved in 100 μL of dimethyl sulfoxide with gentle agitation. The absorbance was measured in each well spectrophotometrically at 570 nm.

### Statistical analysis

One-way analysis of variance (ANOVA) was used to analyze differences among three or more groups. Differences in the means of individual groups were assessed by Bonferroni’s *post hoc* test. Results were considered statistically significant was at p-values < 0.05. Analysis was performed using GraphPad Prism 5 software (La Jolla, CA, USA).

## Supplementary Material

Supplementary Figures
