# Supplementary material for: Anti-inflammatory action of β-hydroxybutyrate via modulation of PGC-1α and FoxO1, mimicking calorie restriction
Source: Aging (Albany NY). 2019 Feb 27;11(4):1283–304. doi: 10.18632/aging.101838 (PMC6402511; doi:10.18632/aging.101838)
Supplement: Supplementary Figures [file aging-11-101838-s001.pdf]

SUPPLEMENTARY FIGURES

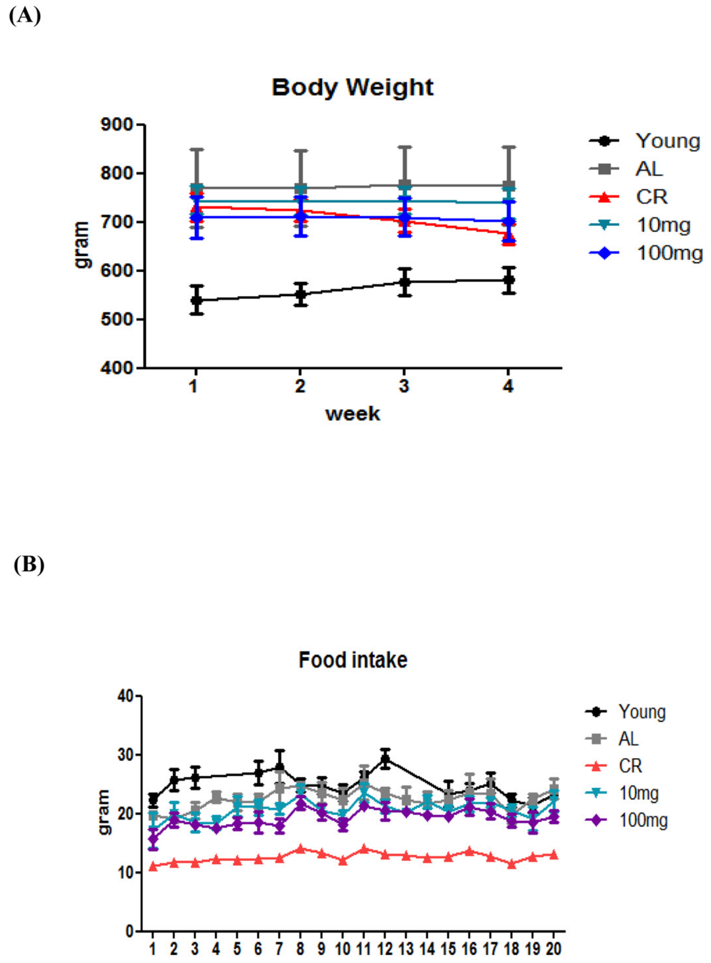

Figure S1. Change of body weight in β-hydroxybutyrate (HB)-treated aged rat. HB were stratified by body weight and randomly assigned to five groups (n = 4). (**A**) Body weight and (**B**) food intake were measured after 30 days of HB treatment. Aged rats were administered 10 or 100 mg of HB.

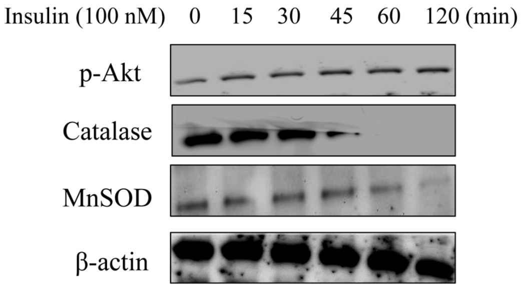

Figure S2. Insulin regulates inflammation in kidney cells. Cytosolic proteins were subjected to western blot analysis for p-Akt, catalase, and MnSOD. HEK293T cell were incubated with or without 100 nM insulin. Three independent experiments were performed, and similar results were obtained.

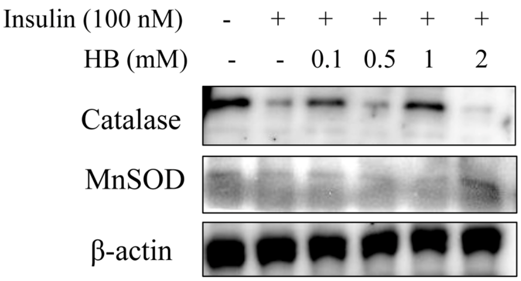

Figure S3. Changes in the levels of anti-inflammatory genes. Cytosolic catalase and MnSOD levels were decreased by pretreatment with 0.1-2 mM β-hydroxybutyrate (HB) for 3 h, followed by incubation with or without 100 nM insulin 100 nM for 6 h.

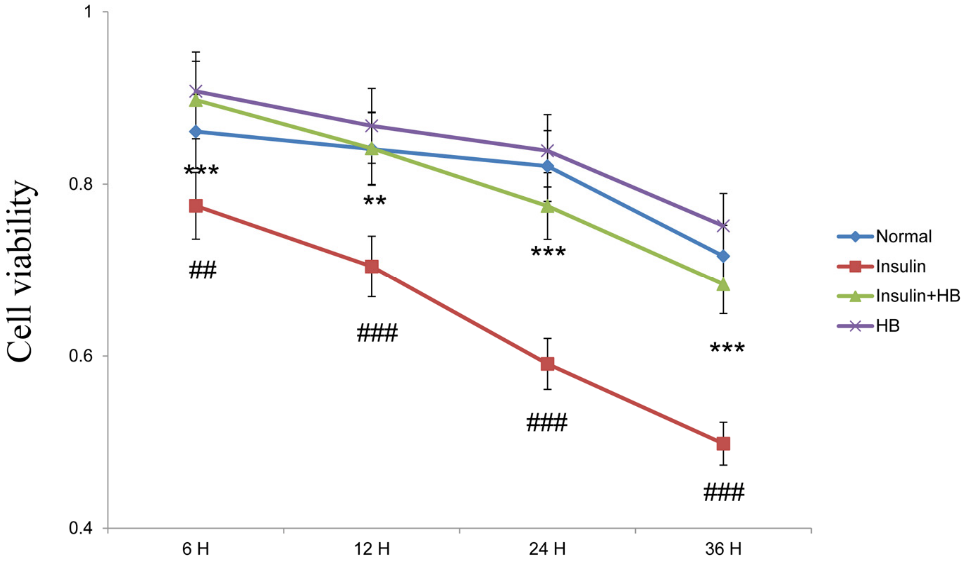

Figure S4. Effects of β-hydroxybutyrate (HB) in HEK293T cells. Cell viability was measured in cells treated with 0.5 mM of HB or 100 nM of insulin. $^{\#}p < 0.05$, $^{\#\#\#}p < 0.001$, vs. non-treated normal; $^{*}p < 0.05$, $^{***}p < 0.001$ vs. insulin treatment.

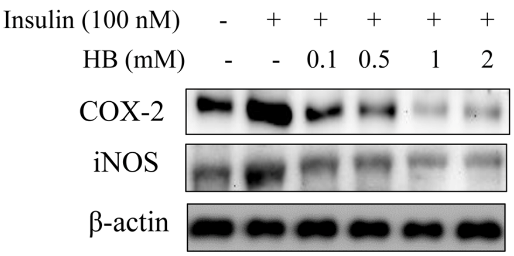

Figure S5. β-hydroxybutyrate (HB) regulates the insulin-induced expression of inflammatory genes. Cytosolic COX-2 and iNOS levels noticeably decreased by pretreatment with 0.1-2 mM HB for 3 h, followed by incubation with or without 100 nM insulin for 6 h.

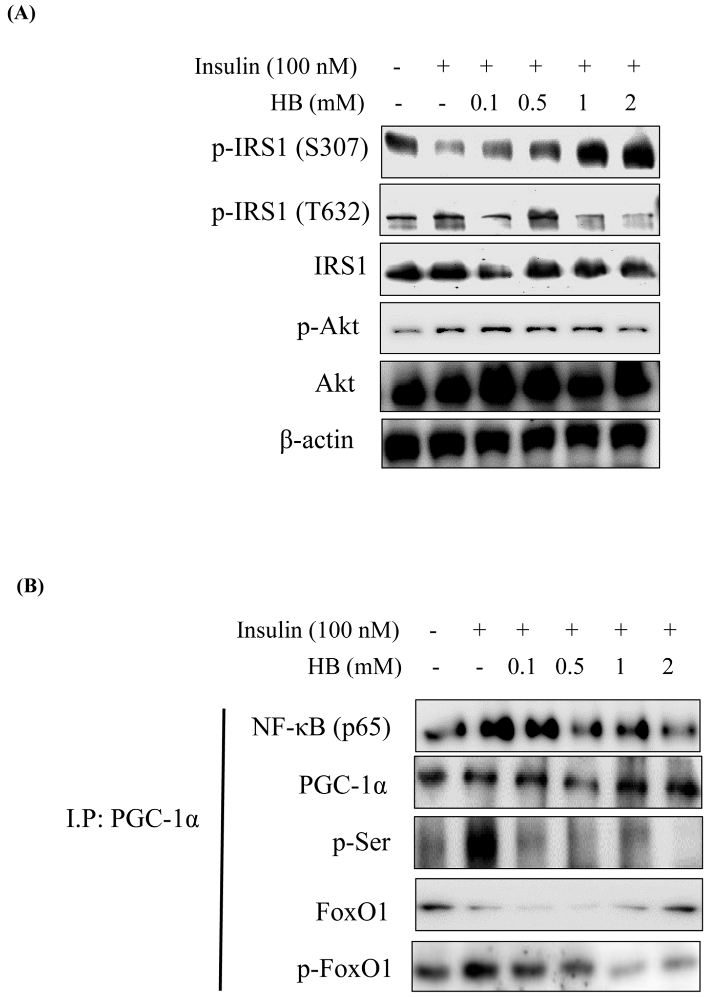

Figure S6. Interaction of PGC-1α between FoxO1 and NF-κB in cells. (**A**) Western blotting was performed to examine the protein levels of p-IRS-1 (Ser307), p-IRS-1 (Tyr632), IRS, p-Akt, and Akt with 0.1–2 mM HB for 3 h, followed by incubation with or without 100 nM insulin for 6 h. (**B**) Western blotting showed that immuno-precipitated PGC-1α were physically associated with p-Serine, PGC-1α, p-FoxO1, FoxO1, and NF-κB, respectively.

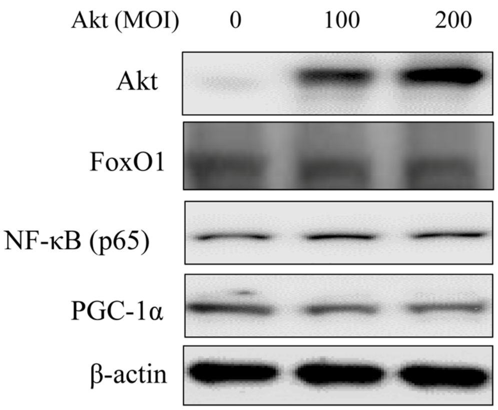

Figure S7. Akt suppressed FoxO1 activity in HEK293T cells. HEK293T cells were pre-transduced with a vector containing Akt (100 and 200 MOI). Protein levels were analyzed by western blotting.

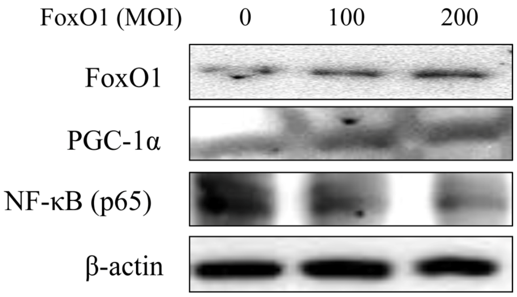

Figure S8. FoxO1 induced inflammation in HEK293T cells. HEK293T cells were pre-transduced with a vector containing FoxO1 (100 and 200 MOI). Protein levels were analyzed by western blotting.

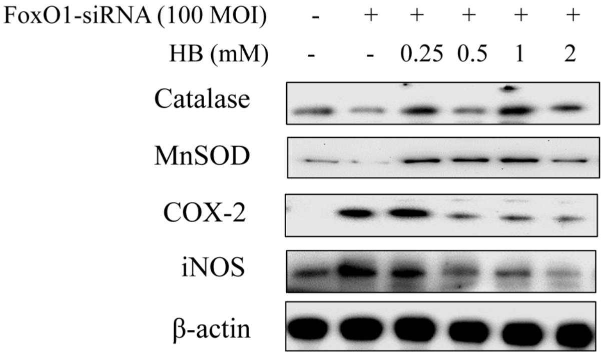

Figure S9. Effect of β-hydroxybutyrate (HB) on the expressions of FoxO1 and NF-κB after FoxO1 knockdown. Western blot analysis was used to assess protein levels in FoxO1 siRNA-treated HEK293T cells. Catalase, MnSOD, COX-2, and iNOS protein levels in cells pretreated for 3 h with HB in the absence or presence of FoxO1 siRNA-transfected cells (200 MOI) for 1 day.
